# Utilization of modern temporary contraceptive methods and its predictors among reproductive-aged women in India: insights from NFHS-5 (2019–21)

**DOI:** 10.3389/fgwh.2023.1219003

**Published:** 2023-10-31

**Authors:** Ritik Agrawal, Manisha Mishra, Tanveer Rehman, Gayathri Surendran, Abhinav Sinha, Srikanta Kanungo, Sanghamitra Pati

**Affiliations:** ^1^ Department of Health Research, ICMR-Regional Medical Research Centre, Chandrasekharpur, Bhubaneswar, India; ^2^ Department of Community Medicine, Christian Medical College, Vellore, India

**Keywords:** family planning, contraceptive pattern, reproductive-aged women, NFHS-5, India

## Abstract

Evidence from various studies on modern contraceptive methods shows that the utilization varies greatly. The present study aimed to estimate the magnitude and determinants for temporary modern contraceptive utilization among reproductive-aged (15-49 years) women in India. We analysed National Family Health Survey-5 data using the “*svyset”* command in STATA software. Modern contraception utilization was estimated using the weighted prevalence, and its correlates were assessed by multivariable regression by reporting an adjusted prevalence ratio (aPR) with 95% confidence interval (CI). QGIS 3.2.1 software was used for spatial analysis of different temporary modern contraceptives. The mean (SD) age of 359,825 respondents was 31.6 (8.5) years with 75.1% (*n* = 270,311) and 49.2% (*n* = 177,165) of them being from rural area and having completed education up to secondary school, respectively. The overall utilization of modern temporary contraception was 66.1% [95%CI: 65.90–66.35, *n* = 237,953]. Multigravida (vs. nulligravida) [aPR = 2.13 (1.98–2.30)], higher education of husband (vs. not educated) [aPR = 1.20 (1.14–1.27)], urban (vs. rural) [aPR = 1.06 (1.03–1.10)], watching television less than once a week (vs. not at all) [aPR = 1.04 (1.01–1.08)], divorced (vs. married) [aPR = 0.65 (0.45–0.94)], and Scheduled Tribe (ST) (vs. unreserved) [aPR = 0.92 (0.88–0.96)] were significant independent determinants. The highest utilization of male condoms, IUCDs, pills and injections were in Himachal Pradesh (86%), Nagaland (64%), Tripura (85%), and Ladakh (20%), respectively. Out of every ten reproductive-aged (15–49 years) women in India, six are using temporary modern contraceptive methods. More intervention strategies should be planned, considering factors like gravida, education, residence, health promotion and caste to attain replacement fertility level.

## Background

Unrestrained population expansion is a persistent global concern. The world's population was approximately 7.7 billion in 2019, and almost one-third were under the age of 15 years (1). The potential for further population growth will be increased as this segment of the population finally reaches reproductive age, with the world population expected to reach 9.7 billion by 2050 (1). India established the first National Family Planning Program in 1952 to limit the population's rapid growth and alleviate poverty (2). However, with a population growth rate of 1.64% and contributing to 18% of the global population, India is far behind the worldwide average of 1.2%. It was anticipated to surpass China as the world's most populous nation by 2027 (3, 4) but in early 2023 India overtook China's population and became the world's most-populated country (5).

The ability to foresee and have the intended family size, spacing between children and timing their births is known as family planning. With the complicated links between fertility, population growth, and poverty, the benefits of family planning for the survival and health of mothers and children are fairly straightforward. In 2000, about 90% of global abortion-related and 20% of obstetric-related mortality and morbidity could have been averted by use of effective contraception by women wishing to postpone or cease further childbearing. A total of 150,000 maternal deaths (representing 32% of all such deaths) could have been prevented with high cost-effectiveness, with much of this benefit reaped in Africa and Asia (6). A crucial part of managing family planning programmes is anticipating demand for contraceptive services (7). This is achieved through the utilization of contraception techniques and addressing unintentional infertility (8). As part of its ongoing effort to broaden the range and accessibility of contraceptive alternatives, India has introduced new contraceptives and provided a full array of family planning services to all segments of the population (9). There are numerous modern methods of contraception available which includes both temporary as well as permanent methods, including the pill, injectables, male and female condoms, emergency contraception, implants, intrauterine contraceptive devices (IUCD), female and male sterilization, and lactational amenorrhea (LAM). Still, various factors influence the decision of a method by an individual, couple, or healthcare professional (10). Traditional family planning approaches, such as the withdrawal method and resorting to abortion, have significant drawbacks. These conventional contraceptive practices often prove ineffective, leading to unintended pregnancies, unsafe abortion procedures, and increased risks to maternal health and wellbeing. Furthermore, they may cause psychological distress, sexual dissatisfaction, and heightened vulnerability to sexually transmitted infections in both women and men (11). Although modern methods have some positive outcomes, they can also have some negative impacts on women's health e.g., pills. Both contemporary and conventional methods of contraception play a role in the overall usage of contraceptives, yet their individual contributions are relatively modest (12).

Female sterilisation is currently the most extensively used method of birth control in India (13). Diminished ovarian function results in a higher incidence of various menstrual irregularities, including menorrhagia, dysmenorrhea, oligomenorrhea, and polymenorrhea. Additionally, women with this condition are more likely to undergo gynaecological surgeries or require a hysterectomy (14). Sterilization also has certain psychological drawbacks, including anxiety and depression symptoms in those with neutral personalities (15), offers no protection from sexually transmitted diseases (STDs), and is difficult and expensive to reverse (16). The average age for female sterilization has been consistent at 26 years, showing little change since the last NFHS survey. Most of the women opting for sterilization reside in rural areas of India and choose this permanent method at a relatively young age. However, the usage of temporary spacing methods is considerably lower in comparison (17). Lack of awareness and fewer opportunities to use modern temporary methods due to unaffordability influence women's decisions to undergo female sterilisation (18).

According to the National Family Health Survey (NFHS-4), approximately 7% of women regretted their choice to undergo sterilization (17). Therefore, the use of temporary contraceptives needs to be addressed, and as many people should appreciate their beneficial effects as possible. Some healthcare benefits of temporary contraceptives like condoms include the fact that they may offer protection from cervical cancer caused by HPV and STDs (14, 19). According to the World Health Organization (WHO), the condom is the only method of contraception that can avert both pregnancy and STDs (18).

Therefore, this study intended to estimate the utilization of temporary modern contraceptives among women of reproductive age in India, determine the associated factors, and assess the geographical distribution of different modern contraceptive utilization using data from the most recent and fifth National Family Health Survey (NFHS-5) 2019-2021.

## Methods

### Study setting

India is the world's most populous country (1.3 billion population), with 28 states and eight union territories (UTs). Each state and UT are further divided into districts. Districts are subdivided into census enumeration blocks and wards in urban areas and villages/taluk in rural areas. The Indian government currently offers a variety of options for family planning and has taken several measures for population control. Assam, Bihar, Chhattisgarh, Jharkhand, Madhya Pradesh, and Rajasthan were the first seven states for which Mission Parivar Vikas was prioritised to substantially increase access to contraceptives and family planning services in high fertility districts with a total fertility rate (TFR) of three and above. Home delivery of contraceptives by health workers at the doorstep of beneficiaries to ensure spacing in births is practised in every state. “360-degree media campaigns” through television (TV) advertisements, posters and promotional banners, and radio shows are provided throughout the year to sensitize people and generate awareness of the need for population control (20).

### Study design and study population

We analysed the NFHS-5 dataset as a part of this study. To start, we submitted our proposal to the Demographic Health Survey (DHS) and, once approved, we were granted permission to access and use the data. NFHS surveys capture data on the health and welfare of the Indian population through a nationally representative sample. We included female participants between the ages of 15 and 49 years who have ever had sex and had not undergone female sterilisation. We focused on women since they utilized the majority of contraceptive techniques. Reproductive age (child-bearing age) women were considered to evaluate modern contraception because contraceptive methods are predominantly used for inducing spacing between pregnancies.

### Sample size and sampling technique

Villages and census enumeration blocks were chosen from districts in rural and urban areas, respectively, through a two-stage sampling procedure. Data collection was done using CAPI (Computer-assisted personal interview) from June 2019 to April 2021 with an inbuilt schedule and proper maintenance of confidentiality of respondents' answers. NFHS-5 methodology, including selecting households and data collection procedures, has been meticulously described and published elsewhere (21). Among women of the reproductive age group, 724,115 women completed the questionnaire, and 364,290 participants did not meet our eligibility criteria, so they were excluded from this study. The selection of the study participants is given in detail in Supplementary File S1.

### Data variables and data sources

We have selected specific variables for analysis based on their potential influence and their observed associations with the utilization of modern temporary contraceptives. The independent variables for assessing the utilization of different modern temporary contraceptive methods were socio-demography and behavioural and family characteristics. Some of the covariates are age (categorized as 15–19 years, 20–24 years, 25–29 years, 30–34 years, 35–39 years, and 40–49 years); respondent's occupation (classified into employed and non-employed based on their working status; those who were categorized under professional/technical/ managerial/clerical/sales/services/household and domestic/agricultural/skilled and unskilled manual were considered as in the employed category and those who were under the not working category were categorized in the non-employed category); and education (completed years of schooling and represented as “no education”: those who had no formal education, “primary”-<5 years of schooling, “secondary”- 5-9 years of education, “higher”-≥10 years of schooling). Exposure to mass media has also been considered for evaluating the knowledge regarding modern contraceptives in the form of three variables: frequency of reading newspapers or magazines, frequency of listening to the radio and frequency of watching TV. This is further sub-grouped into three responses: “not at all”, “less than once a week”, and “at least once a week”. Modern contraceptive utilization was assessed in the survey based on “yes” or “no” responses to the question “have you ever used anything or tried to delay or avoid getting pregnant?”. The modern contraceptive methods used in NFHS-5 are—contraceptive pills, implants, injectables, intrauterine devices (IUDs/ PPIUDs), male condoms, female condoms, male and female sterilization, diaphragm, foam/jelly, the standard day's method, lactational amenorrhoea, and emergency contraception.

### Statistical analysis

STATA 14.2 (Stata Corp, College Station, Texas, USA) was used for statistical analysis. Before analysing, all flagged, missing, and no information cases were removed during the recoding of variables. The NFHS sampling weights were used to justify the differential probabilities of participant selection and ensure the validity of our study findings. The “s*vyset*” command was used to declare the dataset as survey type and to estimate the population's weighted proportion. Utilizing the “svyset” command in Stata is indispensable when dealing with complex survey data. It plays a vital role in allowing Stata to effectively consider survey design elements such as stratification, clustering, and varying selection probabilities. Neglecting to employ “svyset” may lead to distorted estimates and imprecise standard errors, underscoring its significance in ensuring the accuracy of analyses in complex survey research. Modern contraception utilization across predictors was estimated using the weighted prevalence and reported with 95% confidence interval (CI). Univariate log-binomial regression was done for all the independent variables with the outcome and reported an unadjusted prevalence ratio (PR) with 95% CI.

Consequently, multivariable regression was done after checking for collinearity among the variables using the variance inflation factor and reported adjusted PR with 95% CI. The “*svy linearized: poisson*” command was used for Poisson generalized linear model and adjusting for sampling weights to assess the independent effects influencing the usage of temporary modern contraception. The variable which are found to have collinearity like respondents' educational status, religion, age of marriage, age at first childbirth, number of living children, frequency of reading newspaper or magazine, frequency of listening to radio and decision makers on contraceptive use were removed from the regression analysis. Variables with *p* values less than 0.05 were considered statistically significant in the final model. To determine the distribution of the four most used temporary modern contraception (pills, IUCD, condoms, and injections) in India, we used QGIS 3.2.1 software. To represent it nationally, we used the weighted prevalence.

### Ethical considerations

There is no risk to participants because the current study is based on secondary, anonymized data obtained from DHS. Informed consent for all the respondents was obtained during the survey. The dataset used is duly acknowledged and cited wherever needed.

## Results

The analysis included findings from 359,825 women aged 15–49 years who ever had sex and had not undergone female sterilisation. The socio-demographic characteristics of the surveyed women are shown in Table 1. The respondents' mean (SD) age was 31.58 ± 8.48 years. Most of them were unemployed (*n* = 37,728, 71.26%), and were rural residents (*n* = 270,311, 75.12%). Concerning educational status, 49.24% of the women (*n* = 177,165) and 54.31% of their husbands (*n* = 28,452) had completed their education up to secondary. The behavioural and family characteristics of the participants are shown in Table 2. Around 31.34% (*n* = 51,323) had had sexual intercourse, and 14.27% (*n* = 43,161) had their first childbirth before reaching 18 years of age. Of total, 30.44% (*n* = 109,531) and 86.23% (*n* = 310,278) did not watch TV and radio, respectively.

**Table 1:** Utilization of modern contraceptives across socio-demographic characteristics among reproductive age group women covered in the NFHS-5 (*N* = 359,825).

Characteristics and categories	Frequency, %^a^	Weighted frequency, %^a^	Utilization of modern contraception^b^
Age of respondent (in years)			
15–19	16,909 (4.70%)	19,108 (5.31%)	8,268 (43.27%)
20–24	67,527 (18.77%)	71,799 (19.95%)	42,254 (58.85%)
25–29	82,302 (22.87%)	82,970 (23.06%)	57,466 (69.26%)
30–34	62,980 (17.50%)	61,311 (17.04%)	45,114 (73.58%)
35–39	52,579 (14.61%)	49,813 (13.84%)	35,993 (72.26%)
40 and above	77,528 (21.55%)	74,824 (20.79%)	48,857 (65.30%)
Caste (*n* = 336,253)			
Scheduled caste	65,882 (19.59%)	76,193 (22.66%)	49,784 (65.34%)
Scheduled tribe	72,532 (21.57%)	32,469 (9.66%)	19,237 (59.25%)
Other backward class	128,448 (38.20%)	148,591 (44.19%)	97,200 (64.74%)
General category & others	69,391 (20.64%)	79,000 (23.49%)	56,266 (71.22%)
Respondent's educational status			
No education	86,231 (23.96%)	83,660 (23.25%)	53,177 (63.56%)
Primary	44,890 (12.48%)	43,495 (12.09%)	29,039 (66.76%)
Secondary	177,165 (49.24%)	172,901 (48.05%)	114,922 (66.47%)
Higher	51,539 (14.32%)	59,768 (16.61%)	40,815 (68.29%)
Respondent's occupation (*n* = 52,944)			
Employed	15,216 (28.74%)	13,481 (25.46%)	8,816 (65.40%)
Non-employed	37,728 (71.26%)	39,463 (74.54%)	27,402 (69.44%)
Residence			
Rural	89,514 (24.88%)	116,797 (32.46%)	156,799 (64.52%)
Urban	270,311 (75.12%)	243,028 (67.54%)	81,154 (69.48%)
Wealth index (*n* = 3,62,421)			
Poorest	80,440 (22.36%)	72,074 (20.03%)	45,953 (63.76%)
Poorer	79,446 (22.08%)	71,680 (19.92%)	46,888 (65.41%)
Middle	70,009 (19.46%)	68,494 (19.04%)	43,467 (63.46%)
Richer	65,712 (18.26%)	71,396 (19.84%)	46,088 (64.55%)
Richest	64,218 (17.85%)	76,180 (21.17%)	55,557 (72.93%)
Religion (*n* = 3,54,284)			
Hinduism	255,736 (72.18%)	228,719 (78.67%)	183,622 (65.88%)
Islam	52,848 (14.92%)	58,752 (16.58%)	40,454 (68.86%)
Christianity	31,331 (8.84%)	7,666 (2.16%)	4,008 (52.28%)
Sikhism	8,986 (2.54%)	6,525 (1.84%)	4,666 (71.51%)
Buddhism	4,961 (1.40%)	1,890 (0.53%)	1,060 (56.09%)
Jainism	422 (0.12%)	732 (0.21%)	555 (75.85%)
Current marital status			
Never married	8,687 (2.41%)	6,793 (1.89%)	3,683 (54.21%)
Married	331,652 (92.17%)	334,396 (92.93%)	226,318 (67.68%)
Widowed	12,681 (3.52%)	12,128 (3.37%)	5,713 (47.10%)
Divorced	2,522 (0.70%)	2,232 (0.62%)	702 (31.46%)
Separated	4,283 (1.19%)	4,276 (1.19%)	1,537 (35.95%)
Age of marriage (*n* = 2,278)			
<18 years	1,453 (63.78%)	1,598 (70.15%)	726 (45.45%)
≥18 years	825 (36.22%)	680 (29.85%)	275 (40.49%)
Husband's education (*n* = 52,386)			
No education	8,387 (16.01%)	8,043 (15.35%)	4,912 (61.08%)
Primary	6,613 (12.62%)	6,816 (13.02%)	4,720 (69.24%)
Secondary	28,452 (54.31%)	27,587 (52.66%)	19,145 (69.40%)
Higher	8,934 (17.05%)	9,940 (18.97%)	7,133 (71.76%)

^a^ Column percentage.

^b^ Row percentage.

**Table 2:** Utilization of modern contraceptive use across behavioural and family characteristics among reproductive age group women covered in the NFHS-5 (*N* = 359,825).

Characteristics and categories	Frequency (%)^a^	Weighted Frequency (%)^a^	Utilization of modern contraception^b^
Age at first sex (in years) (*n* = 163,764)			
Less than 18 years	51,323 (31.34%)	56,869 (34.73%)	36,026 (63.35%)
18–24	96,816 (59.12%)	94,345 (57.61%)	61,401 (65.08%)
25 and above	15,625 (9.54%)	12,550 (7.66%)	7,675 (61.16%)
Age at first childbirth (in years) (*n* = 302,401)			
Less than 18 years	43,161 (14.27%)	46,201 (15.28%)	31,769 (68.76%)
18–24	201,813 (66.74%)	202,835 (67.08%)	148,834 (73.38%)
25 and above	57,427 (18.99%)	53,364 (17.65%)	38,023 (71.25%)
Total children ever born			
No child	57,424 (15.96%)	59,553 (16.55%)	20,865 (35.04%)
One child	93,539 (26.00%)	99,478 (27.65%)	66,234 (66.58%)
Two children	103,231 (28.69%)	101,809 (28.29%)	76,818 (75.45%)
Three and above	105,631 29.36%)	98,985 (27.51%)	74,036 (74.79%)
Number of living children			
No child	59,918 (16.65%)	62,124 (17.27%)	21,804 (35.10%)
One child	97,712 (27.16%)	103,835 (28.86%)	69,639 (67.07%)
Two children	106,321 (29.55%)	104,914 (29.16%)	79,729 (75.99%)
Three and above	95,874 (26.64%)	88,952 (24.72%)	66,781` (75.08%)
Frequency of reading newspaper or magazine			
Not at all	254,919 (70.85%)	246,272 (68.44%)	160,448 (65.15%)
Less than once a week	63,654 (17.69%)	65,556 (18.22%)	44,808 (68.35%)
At least once a week	41,252 (11.46%)	47,997 (13.34%)	32,697 (68.12%)
Frequency of listening to the radio			
Not at all	310,278 (86.23%)	313,077 (87.01%)	206,862 (66.07%)
Less than once a week	34,882 (9.69%)	32,627 (9.07%)	21,823 (66.89%)
At least once a week	14,665 (4.08%)	14,121 (3.92%)	9,268 (65.63%)
Frequency of watching TV			
Not at all	109,531 (30.44%)	105,433 (29.30%)	67,202 (63.74%)
Less than once a week	78,752 (21.89%)	72,215 (20.07%)	48,889 (67.70%)
At least once a week	171,542 (47.67%)	182,177 (50.63%)	121,862 (66.89%)
Decisions make on contraceptive use (*n* = 156,054)			
Mainly respondent	15,747 (10.09%)	16,025 (10.27%)	15,998 (99.84%)
Mainly husband, partner	13,618 (8.73%)	12,772 (8.18%)	12,739 (99.74%)
Joint decision	126,689 (81.18%)	127,257 (81.55%)	127,032 (99.82%)

^a^ Column percentage.

^b^ Row percentage.

### Overall utilization of temporary modern contraceptives

The overall utilization of temporary contraceptives was 66.13% [(95%CI: 65.90-66.35), *n* = 237,953]. It was highest in Delhi (86.13%), Tripura (85.11%) and Odisha (83.98%). The overall statewide weighted prevalence of modern contraceptives is given in detail in the Supplementary File S2. In the age group of 30-34 years, 73.58% of women used modern contraceptive methods. Utilization was higher among urban (69.48%), married (67.68%), and higher educated (68.29%) individuals (Tables 1, 2). The most commonly used method was male condoms (52.42%) as the primary mode of contraception, followed by pills (27.51%). Details are given in Supplementary File S3.

**Figure 1:**
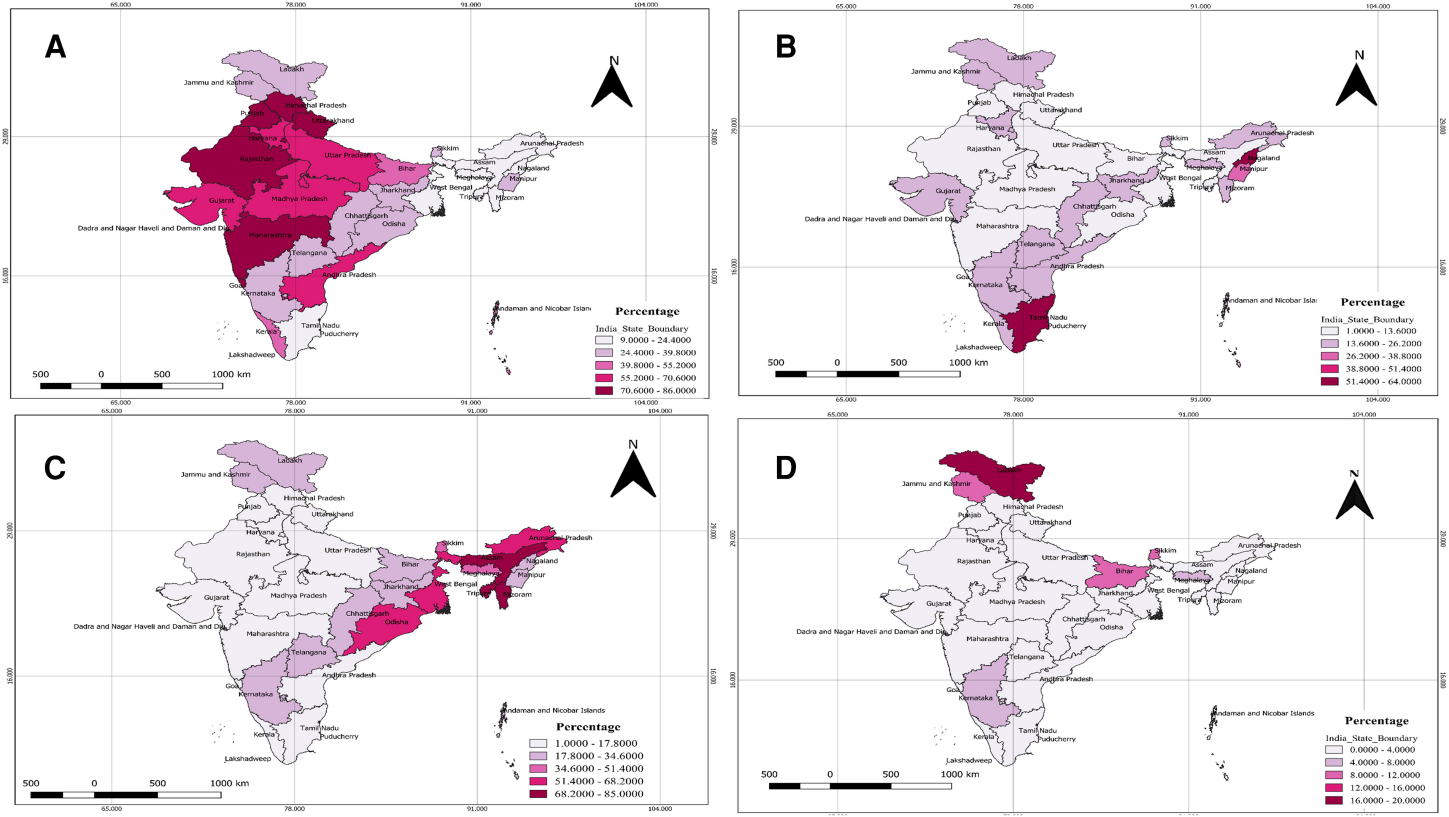
Utilization of different commonly used temporary contraceptive methods across the states and UTs of India. (**A**) Statewide distribution of utilization of male condoms among reproductive-aged women in India. (**B**) Statewide distribution of utilization of IUCD among reproductive-aged women in India. (**C**) Statewide distribution of utilization of pills among reproductive-aged women in India. (**D**) Statewide distribution of utilization of injections among reproductive-aged women in India.

Figures 1A–D shows the utilization of different commonly used temporary contraceptive methods across the states and UTs of India. The pattern of male condom utilization in Figure 1A indicates that it is more prominent in the central, western, and some parts of the northern region, with the highest in Himachal Pradesh (86%) and Chandigarh (85%), followed by Punjab & Uttarakhand (82%). Similarly, the use of IUCD (Figure 1B) was highest in Nagaland (64%) and Tamil-Nadu (63%), followed by Manipur (34%) & Ladakh (26%). Figure 1C shows the usage of pills in northeastern and some eastern regions, with the highest in Tripura (85%), followed by Assam (76%) and Mizoram (72%). Figure 1D shows the highest use of injection in Ladakh (20%), followed by Bihar (12%) & Jammu and Kashmir (11%).

### Determinants of contraceptive use

Predictors of utilization of modern contraception are described in Table 3. We removed the variables found to be collinear (respondent's educational status, religion, age at marriage, age at first childbirth, number of living children, frequency of reading newspaper, frequency of listening to radio, and decision making on contraceptive use) from the multivariable regression model. Respondents who belonged to scheduled tribes (STs) were 8% less [aPR = 0.92 (0.88-0.96)] likely to use modern contraceptives as compared with the general category. The utilization of modern contraception was 3% [aPR = 1.03 (1.00–1.06)] more among unemployed than employed women. The usage of modern contraceptives was 6% [aPR = 1.06 (1.03-1.10)], more common among those women who resided in urban areas than their rural counterparts. Regarding marital status, divorced women used 35% less [aPR = 0.65 (0.45–0.94)] modern contraceptives than married women. The use of modern temporary methods among women whose husband had completed higher education was 1.2 times [aPR = 1.20 (1.14–1.27)] the prevalence among women whose husbands were not educated. The utilization of modern contraception was twice [aPR = 2.13 (1.98–2.30)] more among women with three or more children than those without children. Women who watched TV less than once a week were 1.04 times [aPR = 1.04 (1.01–1.08)] more likely to use modern contraceptives than those who do not watch TV at all. The use of temporary modern contraceptive methods among women in the fourth wealth quintile (“richer”) was 0.94 times [aPR = 0.94 (0.89–0.99)] the prevalence among women in the poorest wealth quintile.

**Table 3:** Determinants of modern contraceptives utilization among reproductive age group women covered in the NFHS-5 (*N* = 359,825).

Characteristics	PR (95% CI)	*p*-value	Adjusted PR (95% CI)	*p*-value
Age of respondent (in years)				
15–19	Reference		Reference	
20–24	1.36 (1.32–1.39)	<0.001	0.94 (0.85–1.03)	0.212
25–29	1.60 (1.56–1.63)	<0.001	0.99 (0.90–1.09)	0.892
30–34	1.70 (1.65–1.74)	<0.001	1.02 (0.93–1.13)	0.588
35–39	1.66 (1.62–1.71)	<0.001	0.98 (0.88–1.08)	0.716
40 and above	1.50 (1.47–1.54)	<0.001	0.94 (0.85–1.03)	0.235
Caste*				
Scheduled caste	0.91 (0.90–0.92)	<0.001	0.96 (0.92–1.00)	0.079
Scheduled tribe	0.83 (0.82–0.84)	<0.001	0.92 (0.88–0.96)	0.001
Other backward class	0.91 (0.90–0.92)	<0.001	0.93 (0.90–0.96)	<0.001
General category & others	Reference		Reference	
Respondent's educational status^a^				
No education	Reference	-	-	
Primary	1.05 (1.03–1.06)	<0.001	-	-
Secondary	1.04 (1.03–1.05)	<0.001	-	-
Higher	1.07 (1.06–1.08)	<0.001	-	-
Respondent's occupation*				
Employed	Reference		Reference	
Non-employed	1.06 (1.04–1.08)	<0.001	1.03 (1.00–1.06)	0.014
Residence*				
Urban	1.07 (1.06–1.08)	<0.001	1.06 (1.03–1.10)	< 0.001
Rural	Reference		Reference	
Wealth index*				
Poorest	Reference		Reference	
Poorer	1.02 (1.01–1.03)	<0.001	1.00 (0.97–1.04)	0.661
Middle	0.99 (0.98–1.00)	0.393	0.94 (0.90–0.98)	0.011
Richer	1.01 (1.00–1.02)	0.029	0.94 (0.89–0.99)	0.014
Richest	1.14 (1.13–1.15)	<0.001	0.97 (0.92–1.02)	0.229
Religion^a^				
Hinduism	1.26 (1.22–1.29)	<0.001	-	-
Islam	1.31 (1.28–1.35)	<0.001	-	-
Christianity	Reference	-	-	
Sikhism	1.36 (1.32–1.41)	<0.001	-	-
Buddhism	1.07 (0.98–1.16)	0.093	-	-
Jainism	1.45 (1.32–1.58)	<0.001	-	-
Current marital status*				
Never Married	0.80 (0.77–0.82)	<0.001	1.02 (0.57–1.81)	0.940
Married	Reference		Reference	
Widowed	0.69 (0.67–0.71)	<0.001	0.73 (0.65–0.81)	<0.001
Divorced	0.46 (0.41–0.52)	<0.001	0.65 (0.45–0.94)	0.024
Separated	0.53 (0.50–0.56)	<0.001	0.80 (0.67–0.96)	0.015
Age of marriage (in years)^a^				
<18 years	1.12 (0.97–1.28)	0.097	-	-
≥18 years	Reference	-	-	
Husband's education*				
Not educated	Reference	Reference		
Primary	1.13 (1.09–1.17)	<0.001	1.13 (1.07–1.18)	<0.001
Secondary	1.13 (1.10–1.16)	<0.001	1.16 (1.11–1.21)	<0.001
Higher	1.17 (1.14–1.21)	<0.001	1.20 (1.14–1.27)	<0.001
Age at first sex (in years)				
Less than 18 years	1.03 (1.01–1.05)	0.003	0.97 (0.92–1.03)	0.435
18–24	1.06 (1.04–1.08)	<0.001	1.02 (0.96–1.07)	0.431
25 and above	Reference	Reference		
Age at first childbirth (in years)^a^				
Less than 18 years	Reference	-	-	
18–24	1.06 (1.05–1.07)	<0.001	-	-
25 and above	1.03 (1.02–1.04)	<0.001	-	-
Total children ever born*				
No child	Reference	Reference		
One child	1.90 (1.86–1.93)	<0.001	1.93 (1.80–2.07)	<0.001
Two children	2.15 (2.11–2.18)	<0.001	2.07 (1.92–2.22)	<0.001
Three and above	2.13 (2.09–2.17)	<0.001	2.13 (1.98–2.30)	<0.001
Number of living children^a^				
No child	Reference	-	-	
One child	1.91 (1.87–1.94)	<0.001	-	-
Two children	2.16 (2.13–2.20)	<0.001	-	-
Three and above	2.13 (2.10–2.17w)	<0.001	-	-
Frequency of reading newspaper or magazine^a^				
Not at all	Reference	-	-	
Less than once a week	1.05 (1.03–1.05)	<0.001	-	-
At least once a week	1.04 (1.03–1.05)	<0.001	-	-
Frequency of listening to the radio^a^				
Not at all	1.00 (0.98–1.02)	0.465	-	-
Less than once a week	1.01 (0.99–1.04)	0.078	-	-
At least once a week	Reference	-	-	
Frequency of watching TV*				
Not at all	Reference		Reference	
Less than once a week	1.06 (1.05–1.07)	<0.001	1.04 (1.01–1.08)	0.012
At least once a week	1.04 (1.04–1.05)	<0.001	1.03 (0.99–1.06)	0.078
Decision makers on contraceptive use^a^				
Mainly respondent	1.00 (0.99–1.00)	0.195	-	-
Mainly husband, partner	Reference	-	-	
Joint decision	1.00 (0.99–1.00)	0.181	-	-

PR, Prevalence ratio (PR); aPR, adjusted prevalence ratio.

^a^ Variables not included in the multivariable analysis because of collinearity.

* *p*-value < 0.05.

## Discussion

The overall utilization of temporary contraception among reproductive-aged (15–49 years) women in India based on secondary data analysis of NFHS-5 data (2019–21) was 66.1% [95%CI: 65.90–66.35, *n* = 237,953]. Multigravida (vs. nulligravida), higher education of husband (vs. not educated), urban residence (vs. rural), watching TV less than once a week (vs. not at all), unemployed (vs. employed), divorced (vs. married), and ST (vs. general and others) were significant independent determinants. The highest utilization of male condoms, IUCDs, pills and injections were in Himachal Pradesh (86%), Nagaland (64%), Tripura (85%), and Ladakh (20%), respectively.

Aggressive and forceful male sterilisation campaigns during the seventeenth century stigmatized the method entirely and burdened female sterilisation (15). Family planning programs in India have been traditionally intended to promote permanent ways of preventing pregnancy with the concern of the need to restrict the rising population trajectory (2). This resulted in an invariably explicit promotion of sterilization, with the main focus exclusively on women. As of the current scenario, female sterilization, in particular, is the most commonly used method of contraception in India (16). As of 2011, statistics from the United Nations indicated that India was accountable for a significant 36% of all global female sterilization cases (22). A 2021 study by Singh et al. revealed variations in the reliance on female sterilization across India. In the southern region, over 80% of women have been choosing sterilization for more than two decades, possibly due to strong demand for family size limitation and better access to family planning services. High prevalence in the south also relates to societal taboos around temporary alternatives, lack of awareness, incentives for sterilization, and unequal male involvement in contraception (23). De Oliveira et al. suggested that women from disadvantaged and marginalized groups continue to have sterilisation as a preferred method of contraception compared with reversible methods (2). Since then, the government's policy has evolved, as shown by the Family Planning 2020 action plan, that continues to support sterilisation while simultaneously promoting modern reversible contraceptives (13).

Our study found a higher prevalence of temporary modern contraceptives than an earlier study conducted using NFHS-3 data (34%) and in Liberia (23.87%) (2, 24). Ejembi et al. suggested that the utilization of modern contraceptives in Nigeria (21.6%) was lower than that of the temporary methods in our study (25). However our result is lower than the findings of another study, which found that the reported use of temporary contraceptive methods was 78.8% (14). Bangladesh (62.4%) and Tanzania (34.3%) revealed a lower prevalence of overall modern contraceptive utilization than temporary modern contraceptive utilization in the current study (26, 27). Andia et al. conducted a study in Uganda and found that the prevalence of barrier methods was high (82%) (28). These differences could be because of sociodemographic and behavioural characteristics. Additionally, personal knowledge, awareness, information, understanding of modern contraceptives, and sociocultural differences between various countries could significantly impact contraceptive use, which could cause discrepancies. Furthermore, the time interval between earlier studies and this study differs.

Women residing in cities were more likely to use modern contraception than those living in rural areas. Such results align with the research conducted in Afghanistan, Nigeria, Bangladesh, and Ethiopia, which showed that urban resident women are more likely than rural women to utilize modern contraceptives (15, 16, 29, 30). This might be because urban individuals are more educated, earn relatively more money, and have easier access to medical facilities and mass media than rural women, all of which positively impact the use of modern contraceptives. Furthermore, rural women require more children to assist them with fieldwork, reducing the conviction to have smaller families and reducing the use of contraceptives (30, 31, 32, 33).

A previous study found a positive correlation between a husband's education and contraceptive use which is in line with our study findings, suggesting that modern contraceptive utilization is substantially proportional to the husband's educational status (29). A husband's education substantially impacted contraceptive use compared to a wife's, particularly for male-dominant methods such as condoms and male sterilization (34). Educated husbands directly influenced better contraceptive knowledge and were more willing to sacrifice their cultural beliefs, perception, and attitude toward temporary methods. Additionally, it also motivates their partner to use contraceptives. Indirectly it is associated with higher income, which has a synergistic effect with contraceptive use (31, 32, 35). Caste was significantly associated with the temporary modern contraceptives' utilization. Our study highlights that utilization was lesser among scheduled tribes (STs) than in the general category. The results are in harmony with the study conducted using NFHS-4 (36). This might be due to less awareness and knowledge of modern contraceptives among tribes because of socio-cultural barriers, including lower access to education and marginalization.

Preceding studies have highlighted that women with three children reported the highest use of modern contraceptives. The lowest utilization was found among women who have no children, which is again in harmony with our findings (37). This implies that women with no or fewer children will try to have more children to attain the intended family size, thus reducing contraceptive use (38). Our findings suggest that unemployed women were significantly associated with contraceptive use. Unemployed women spend most of their time at home, so they utilise more contraceptives to attain desired family size than employed individuals. However, our findings contradict other studies that reported employed women use more contraceptives (39). Contraception usage is lesser among divorced women as compared with married women. Further research is needed in this context to collect more evidence.

Mass media exposure, especially watching TV, is positively associated with using temporary contraceptives. Women who watched TV at least once a week used more contraceptives than those without exposure to mass media. The finding concurs with a study in Nepal and NFHS-4, which found that TV exposure strongly impacts contraceptive usage (36, 40). Ghosh et al. reported that women would become aware of various family planning methods if they access the information offered by different mass media platforms. TV is the most efficient medium for conveying family planning messages, followed by use in conjunction with other mass media formats (36).

### Policy implications

The findings of this study show that the prevalence of contraceptive use is quite close to the actual demand as estimated in a previous study, suggesting that the National Family Planning Program is largely successful in its objective (41). The male condom was the most widely used temporary method, so promotion of and access to other temporary methods might need to be boosted. It is advisable to diversify contraceptive options by actively promoting access to a range of temporary contraceptive methods, thus affording individuals a broader spectrum of family planning choices. Additionally, there is a need to bolster outreach and communication efforts, with a particular focus on informing individuals about the array of available contraceptives and the dynamic nature of family planning policies. This approach aims to counteract potential confusion and the spread of misinformation. Thirdly, enhancing community distribution programs can play a pivotal role in improving rural women's accessibility to contraceptives. This strategy is crucial in bridging the gap between remote communities and healthcare services, ensuring that contraceptives are readily available to those who need them. Additionally, the study highlights the importance of empowering women, particularly through education and comprehensive sex education programs, to enable them to make informed decisions regarding contraceptive usage. Furthermore, there is a pressing need to support unmarried or divorced women in accessing contraceptives without the burden of stigma. Providing non-judgmental healthcare services and conducting sensitization efforts can contribute significantly to this objective. Lastly, the promotion of responsible contraceptive use and accurate family planning information dissemination through strategies such as Information Education Communication (IEC) and Behavioural Change Communication (BCC) is vital.

### Strengths and limitations

The National Family Health Survey-5 (NFHS-5) is a large-scale multi-round survey that gathers information with a high response rate in rural and urban areas throughout all 36 Indian states and UTs, using standard procedures and quality assurance. The current study's use of NFHS data yields reliable national-level estimates. The main strength of our research is this is the first study to estimate the prevalence and determine the correlates of the utilization of temporary modern contraceptives using nationally representative data. It also has a few limitations. The study did not address the relationship of qualitative variables such as socio-cultural factors to current contraceptive use. As a result of being a cross-sectional survey, it does not reveal any differences in the use of temporary modern methods in India by time frame, and the findings should be interpreted cautiously. Behavioural characteristics of men were collected from their partners; instead, the couples should have been asked to obtain accurate information. Analyzing the wealth index associated with the utilization findings was difficult. Because of collinearity we could not assess some of the potential variables which can influence the modern contraceptive utilization. There are some limitations with the NFHS-5 datasets also which include potential sampling bias, reliance on self-reported data, non-response bias etc.

## Conclusion

Out of every ten reproductive-aged (15–49 years) women in India, six are using temporary modern contraceptive methods. More intervention strategies should be planned, considering factors like gravida, education, residence, health promotion and caste to attain replacement fertility level.

## Data Availability

The dataset analyzed for this study is available at the NFHS data repository held at DHS https://www.dhsprogram.com/methodology/survey/survey-display-541.cfm. Further inquiries can be directed to the corresponding authors.
