# Supplementary figures and images for: Utilization of modern temporary contraceptive methods and its predictors among reproductive-aged women in India: insights from NFHS-5 (2019–21)
Source: Front Glob Womens Health. 2023 Oct 31;4:1219003. doi: 10.3389/fgwh.2023.1219003 (PMC10644831; doi:10.3389/fgwh.2023.1219003)

**Supplementary File 1: Selection of study participants**


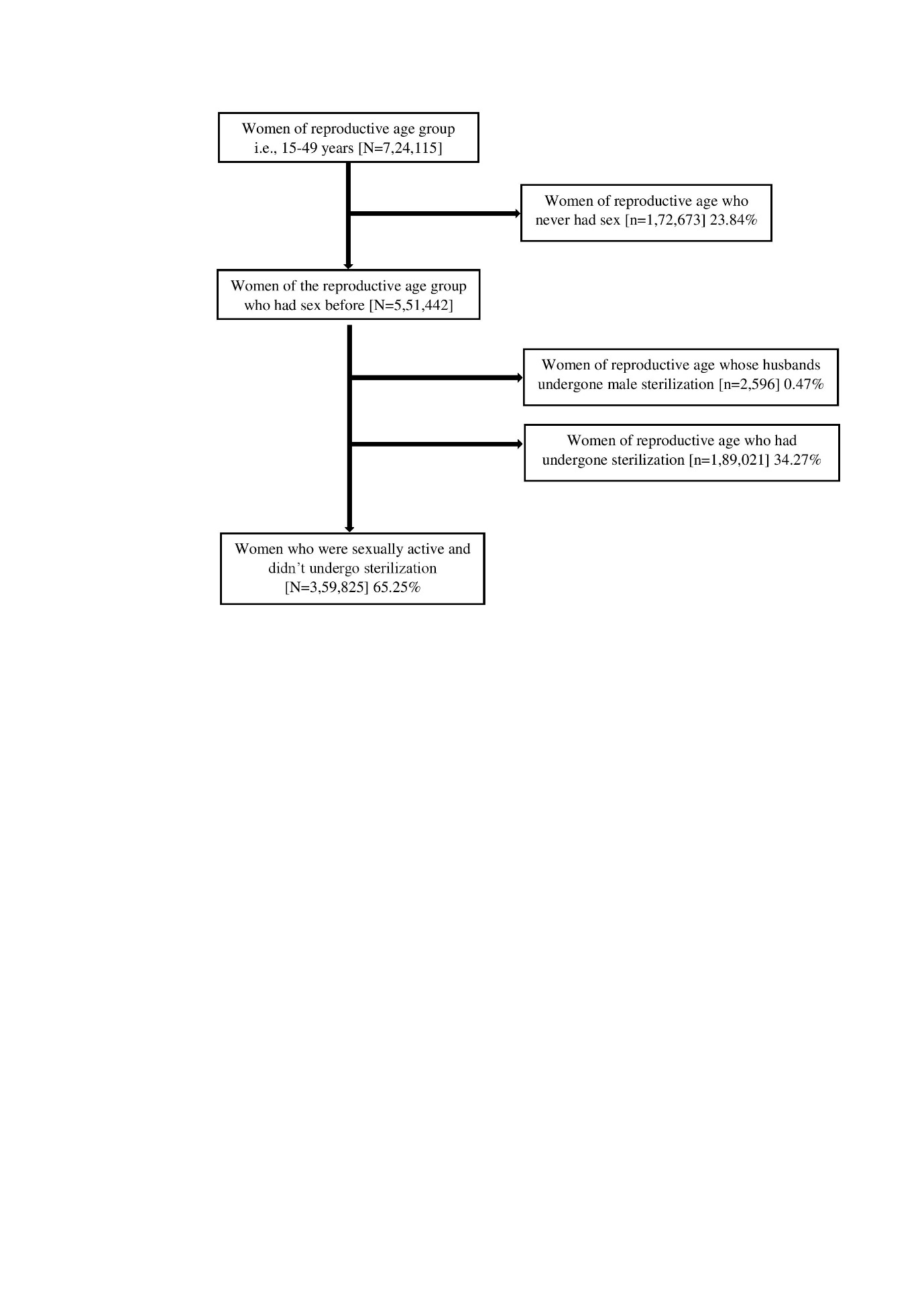

Supplement: Supplementary file 1 [file Datasheet1.docx]
