# Supplementary material for: Utilization of modern temporary contraceptive methods and its predictors among reproductive-aged women in India: insights from NFHS-5 (2019–21)
Source: Front Glob Womens Health. 2023 Oct 31;4:1219003. doi: 10.3389/fgwh.2023.1219003 (PMC10644831; doi:10.3389/fgwh.2023.1219003)
Supplement: Supplementary file 2 [file Datasheet2.docx]

| **S.N.** | **States** | **Using contraceptives** | **Not using contraceptives** |
| --- | --- | --- | --- |
| 1 | Jammu and Kashmir | 2,240 (66.29%) | 1,139 (33.71%) |
| 2 | Himanchal Pradesh | 1,318 (72.35%) | 504 (27.65%) |
| 3 | Punjab | 6,695 (71.27%) | 2,699 (28.73%) |
| 4 | Chandigarh | 291 (82.04%) | 64 (17.96%) |
| 5 | Uttarakhand | 2,742 (81.24%) | 633 (18.76%) |
| 6 | Haryana | 5,419 (72.78%) | 2,027 (27.22%) |
| 7 | Delhi | 5,728 (86.13%) | 923 (13.87%) |
| 8 | Rajasthan | 14,878 (73.57%) | 5,345 (26.43%) |
| 9 | Uttar Pradesh | 59,928 (79.96%) | 15,024 (20.04%) |
| 10 | Bihar | 19,248 (58.55%) | 13,625 (41.45%) |
| 11 | Sikkim | 158 (73.40%) | 57 (26.60%) |
| 12 | Arunachal Pradesh | 280 (69.89%) | 121 (30.11%) |
| 13 | Nagaland | 299 (61.84%) | 184 (38.16%) |
| 14 | Manipur | 752 (83.20%) | 152 (16.80%) |
| 15 | Mizoram | 153 (42.25%) | 208 (57.75%) |
| 16 | Tripura | 1,498 (85.11%) | 262 (14.89%) |
| 17 | Meghalaya | 548 (42.69%) | 735 (57.31%) |
| 18 | Assam | 10,546 (74.11%) | 3,683 (25.89%) |
| 19 | West Bengal | 29,098 (78.83%) | 7,813 (21.17%) |
| 20 | Jharkhand | 5,540 (58.51%) | 3,929 (41.49%) |
| 21 | Odisha | 11,773 (83.98%) | 2,245 (16.02%) |
| 22 | Chattisgarh | 3,717 (55.02%) | 3,040 (44.98%) |
| 23 | Madhya Pradesh | 9,822 (60.55%) | 6,399 (39.45%) |
| 24 | Gujarat | 11,337 (65.24%) | 6,040 (34.76%) |
| 25 | Dadra and Nagar Haveli | 92 (70.89%) | 38 (29.11%) |
| 26 | Maharashtra | 13,998 (49.78%) | 14,121 (50.22%) |
| 27 | Andhra Pradesh | 668 (8.80%) | 6,922 (91.20%) |
| 28 | Karnataka | 6,004 (45.12%) | 7,303 (54.88%) |
| 29 | Goa | 507 (82.35%) | 109 (17.65%) |
| 30 | Lakshadweep | 17 (66.63%) | 8 (33.37%) |
| 31 | Kerala | 4,394 (55.35%) | 3,545 (44.65%) |
| 32 | Tamil Nadu | 6,500 (43.72%) | 8,368 (56.28%) |
| 33 | Puducherry | 117 (48.06%) | 126 (51.94%) |
| 34 | Andaman and Nicobar Islands | 60 (61.79%) | 37 (38.21%) |
| 35 | Telangana | 1,549 (25.95%) | 4,418 (74.05%) |
| 36 | Ladakh | 40 (63.25%) | 23 (36.75%) |
| 37 | Total | 1,21,872 (33.87%) | 2,37,953 (66.13%) |

**Supplementary File 2: Overall statewide prevalence of modern contraceptives utilization among reproductive aged women aged 15-49 in India.**
